# SNX16 is required for hepatocellular carcinoma survival via modulating the EGFR-AKT signaling pathway

**DOI:** 10.1038/s41598-024-64015-6

**Published:** 2024-06-07

**Authors:** Lebin Yuan, Yanqiu Meng, Jiajia Xiang

**Affiliations:** 1 Molecular Centre Laboratory, Jiangxi Medical College, The Second Affiliated Hospital of Nanchang University, Nanchang University, Nanchang, 330006 China; 2 Department of General Surgery, Jiangxi Medical College, The Second Affiliated Hospital of Nanchang University, Nanchang University, Nanchang, 330006 China; 3 Oncology Department, First Affiliated Hospital of Jiangxi Medical College, Nanchang University, Nanchang, 330006 China

**Keywords:** SNX16, HCC, EGFR, Proliferation, Invasion, Cancer, Genetics, Diseases

## Abstract

Sorting nexin 16 (SNX16), a pivotal sorting nexin, emerges in tumor progression complexity, fueling research interest. However, SNX16’s biological impact and molecular underpinnings in hepatocellular carcinoma (HCC) remain elusive. This study probes SNX16’s function, clinical relevance via mRNA, and protein expression in HCC. Overexpression/knockdown assays of SNX16 were employed to elucidate impacts on HCC cell invasion, proliferation, and EMT. Additionally, the study delved into SNX16’s regulation of the EGFR-AKT signaling cascade mechanism. SNX16 overexpression in HCC correlates with poor patient survival; enhancing proliferation, migration, invasion, and tumorigenicity, while SNX16 knockdown suppresses these processes. SNX16 downregulation curbs phospho-EGFR, dampening AKT signaling. EGFR suppression counters SNX16-overexpression-induced HCC proliferation, motility, and invasiveness. Our findings delineate SNX16’s regulatory role in HCC, implicating it as a prospective therapeutic target.

## Introduction

Hepatocellular carcinoma (HCC), prevalent amongst the most malignant hepatic malignancies, accounts for approximately 80% of cases^1^. Despite advancements in therapeutic interventions, including early detection, surgical resection, radiation therapy, liver transplantation, and immunotherapeutic strategies that have significantly augmented patient outcomes, the prognosis remains grim with a two-year survival rate seldom exceeding 50% and a five-year survival probability approximating a meager 10%. Subsequently, the long-term outcome for patients with HCC remains unfavourable^2^. Furthermore, intensive research efforts have concentrated on developing potent targeted therapies for HCC, exemplified by drugs like sorafenib, which specifically target key genes implicated in disease progression^3^, but the exact pathogenesis of HCC is still unclear. Thus, identifying the mechanisms underlying the prognosis of HCC is important.

Sorting nexins (SNXs), a cohort of high-affinity proteins recognizing phosphatidylinositol, are distinguished by a conserved PX domain, vital for their function in membrane trafficking regulation^4^. SNX is involved in transmembrane transport, protein sorting, cellular signalling and organellar trafficking^5^. Escalating evidence underscores the involvement of SNXs in cancer-related biological processes, highlighting their regulatory roles in key signaling pathways underlying oncogenesis^6^. SNX6 has been identified as a promoter of epithelial-mesenchymal transition(EMT), forecasting adverse prognosis, and facilitating the metastatic progression of pancreatic cancer, as emerging research indicates^7^. Moreover, research revelead that SNXs levels expressed have been shown to be tightly coupled to cancer-related EGFR levels^8^. As part of the SNX family, SNX16 has been implicated in hepatitis C virus replication, vesicular K1 inflammatory virus infection, and synaptic growth receptor trafficking^9^. Recent studies have shown that SNX16 has been identified as a cause of a number of cancers, include gastric^10^, ovarian^11^, and bladder cancers^12^. Elevated SNX16 in colorectal cancer fuels disease progression via eEF1A2-driven c-Myc activation^13^. Employing miRNet, a risk-prognostic model centered on the GSEC/miR-101-3p/SNX16/PAPOLG axis, impacting HCC prognosis and immune microenvironment, was established^14^. Yet, SNX16’s explicit role in HCC remains uncharted.

EGFR orchestrates signaling pathways governing cell survival, migration, and growth, central to multiple cellular functions^15,16^. Cumulative evidence suggests a frequent upregulation of EGFR in neoplastic tissues^17^. Early recurrence, metastatic risk, and poor survival are some of the bad clinical outcomes associated with EGFR over-expression and overactivation in HCC^18,19^. Clinical studies for HCC have not shown promising results when using EGFR antibodies and inhibitors^20^, despite their widespread use in cancer patient management^21^. Elevated SNX5 in HCC patients correlates with poor prognosis, facilitating tumor growth and metastasis via activation of the EGFR-ERK1/2 axis^22^. In triple-negative breast cancer, SNX3 emerges as a pivotal regulator, with the EGF-SNX3-EGFR axis shaping tumor progression and metastasis^23^. Meanwhile, the precise modulation of EGFR signaling by SNX16 driving HCC progression remains incompletely elucidated.

Herein, we pioneer an in-depth investigation into the expression profile and functional implications of SNX16 in HCC, both in vitro and in vivo. Our findings unveil unprecedented molecular insights, highlighting SNX16’s critical role in modulating EGFR activation through the AKT pathway, thereby identifying it as a potential therapeutic target and oncogenic player in HCC.

## Results

### Upregulation of SNX16 in human HCC tissue

**Figure 1:**
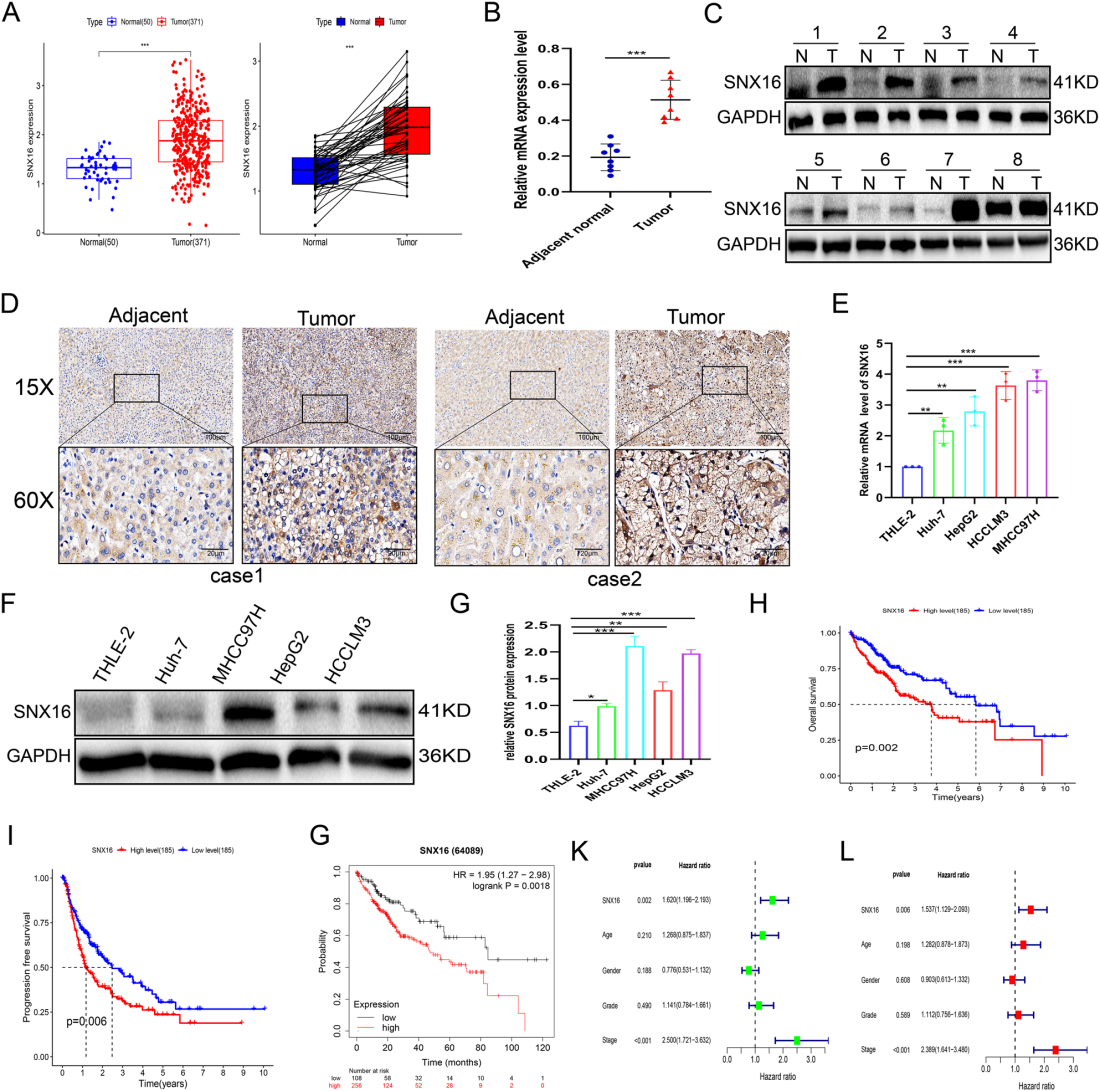
SNX16 is up-regulated and is correlated with poor prognosis in HCC. (**A**-**B**) SNX16 was upregulated and in 371 HCC tissues compared to 50 adjacent non-tumour tissues based on the TCGA-HCC dataset as well as in 50 paired HCC samples than the corresponding normal hepatocellular tissues. Data are expressed as (−log10) for SNX16 expressed (fpkm). The diagram depicts the mean value derived from the dataset. ***P < 0.001. (**C**-**D**) SNX16 expression analysis in 8 HCC tissues and paired normal samples revealed elevated levels at mRNA and protein levels by qPCR and Western blot. ***P < 0.001. (**E**) Immunohistochemistry shows elevated SNX16 protein expression in HCC compared to normal tissue pairs. (**F**-**G**) SNX16 showed higher mRNA and protein expression in human HCC cell lines, particularly MHCC97H and HCCLM3, compared to the normal HCC cell line THLE-2. **P < 0.01, ***P < 0.001. (**H**-**I**) In the TCGA-LIHC cohort, stratifying patients by median SNX16 expression revealed that 185 high-expression cases had significantly diminished overall survival (P = 0.002) and disease-free survival (P = 0.006) compared to 185 low-expression counterparts. (**G**) Kaplan–Meier survival charts of HCC patients demonstrated that patients with higher SNX16 mRNA expression were less likely to survive (P = 0.0018). (**K**-**L**) Univariate Cox analysis identified SNX16 (P = 0.002) and stage (P < 0.001) as significant predictors of overall survival in HCC. Multivariate analysis confirmed SNX16 as an independent prognostic factor for HCC patient outcomes (P = 0.006).

First, to validate the expression of SNX16 in HCC, we analysed the level of SNX16 expression in 371 HCC samples and 50 peritumour samples from TCGA-LIHC dataset. Our findings demonstrated a significant overexpression of SNX16 in hepatocellular carcinoma relative to non-malignant tissues, including matched adjacent normal liver samples (P < 0.001, Fig. 1A). In agreement with prior studies, our IHC, Western (n = 8), and qRT-PCR (n = 8) analyses revealed elevated SNX16 expression in HCC versus adjacent non-tumor tissues (Fig. 1B-D, Supplement Fig. 1A). Endogenous expression levels of SNX16 were also assessed via Western blotting and qRT-PCR in five HCC cell lines, and results consistently demonstrated higher SNX16 expression levels within HCC than normal cell lines THLE-2 (Fig. 1E-G, Supplement Fig. 1B). These observations suggest that SNX16 levels are elevated in HCC and that SNX16 could have specific impacts over various cancer types. Next, we have examined the clinical influence of SNX16 in HCC subjects. Compared to the 185 low expression group, subjects with 185 high SNX16 expression in HCC demonstrated more diminished OS and DFS, according to the TCGA-LIHC cohort (P = 0.002 and P = 0.006 separately, Fig. 1H-I). Kaplan–Meier survival analysis consistently demonstrated that increased SNX16 expression correlated with a significantly reduced OS (P = 0.0018; Fig. 1G). Univariable Cox regression analysis identified advanced stage (HR: 2.500; 95% CI 1.721–3.632; P < 0.001) and elevated SNX16 expression (HR: 1.620; 95% CI 1.196–2.193; P = 0.002) as independent predictors of poor survival outcomes. Multivariate analysis reinforced high SNX16 expression as a prognostic indicator for reduced survival (HR: 1.537, 95% CI 1.129–2.093; P = 0.006; Fig. 1K-L). Furthermore, analysis of 371 cases from TCGA-LIHC dataset correlated high SNX16 mRNA levels with advanced tumor stage (p = 0.016) and T stage (p = 0.012) (Table 1), highlighting its clinical significance. Therefore, these insights imply that SNX16 can be a powerful prognostic factor for patients suffering from HCC.

**Table1:** SNX16 expression and clinicopathological features of 371 liver cancers based on TCGA datebases.

Variables	Clinicopathological characteristics	Numbers	SNX16 low expression	SNX16 high expression	p value
Death/live	live	241	55	75	
	death	260	130	111	
Age	≤ 60	176	85	94	0.349
	> 60	194	100	91	
	unknow	1			
Gender	Female	121	55	65	0.299
	Male	250	129	121	
Stage	I + II	257	138	119	0.016
	III + IV	90	35	55	
	unknow	24			
T	T1 + T2	275	148	127	0.012
	T3 + T4	93	36	57	
	unknow	3			
N	N0	252	127	125	0.622
	N1	4	1	3	
	unknow	115			
M	M0	266	132	134	0.622
	M1	4	3	1	
	unknow	101			

### SNX16 is a positive regulator of HCC cell proliferation

**Figure 2:**
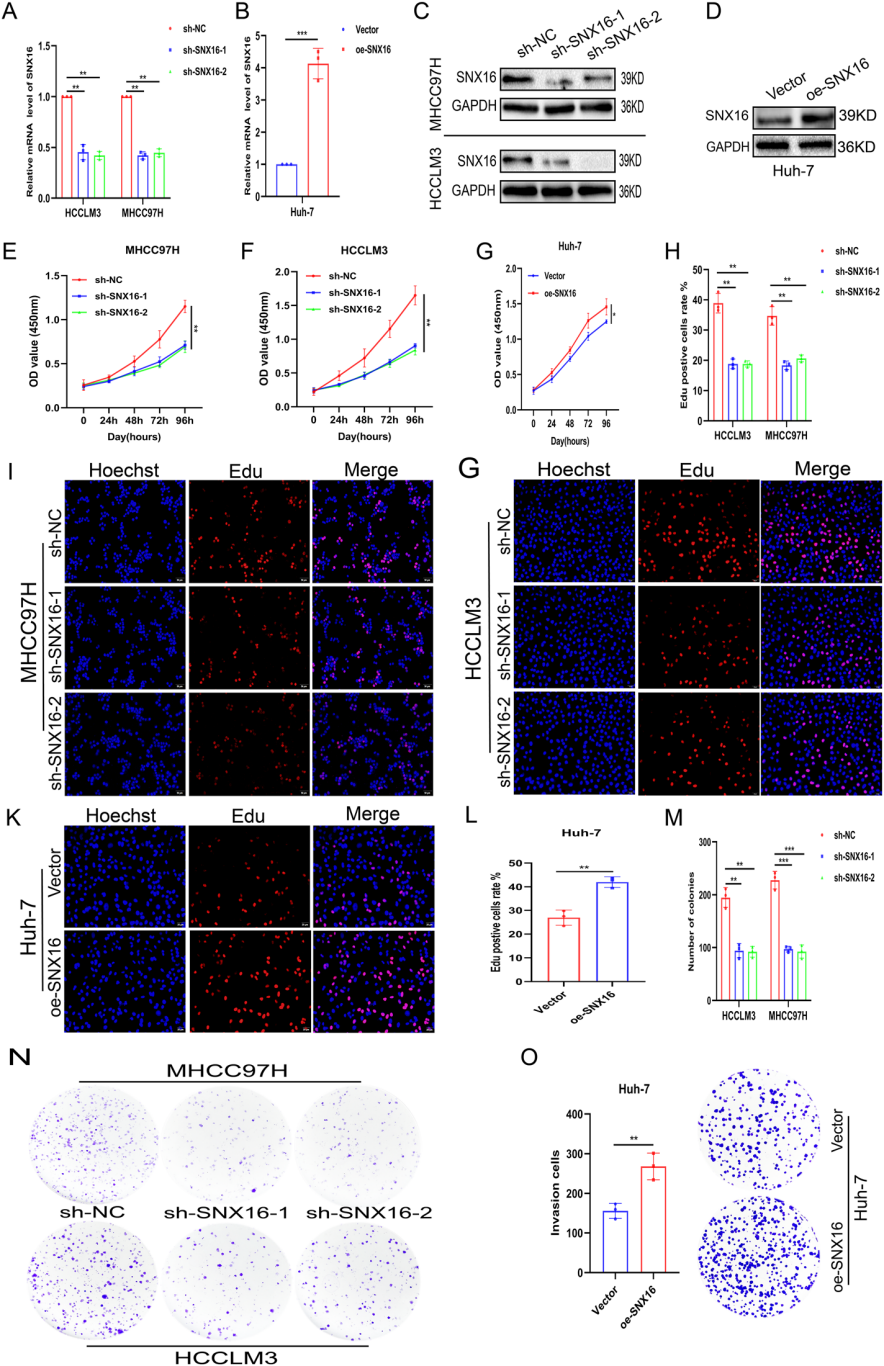
Expression of SNX16 affects the ability of HCC cells to proliferate (**A**-**D**) MHCC97H and HCCLM3 cells underwent knockdown, whereas Huh7 cells were engineered for SNX16 overexpression. Subsequent transcriptomic and proteomic assessments validated the efficacious modulation of SNX16 in each cell model post-transfection. (**E**–**G**) CCK-8 assays showed reduced absorbance post-SNX16 knockdown in MHCC97H and HCCLM3 cells, indicating proliferation suppression. Conversely, SNX16 overexpression in Huh-7 cells increased absorbance, signifying enhanced proliferation. *P < 0.05, **P < 0.01. (**H**–**L**) EdU assays, reflecting DNA replication, revealed decreased EdU-positive cells after SNX16 knockdown in MHCC97H and HCCLM3, and increased positivity upon overexpression in Huh-7 cells. Scale bars: 20 μm, 50 μm. **P < 0.01, ***P < 0.001. (**M**–**O**) Clonogenic assays further reveal that SNX16 depletion reduces colony number and size in MHCC97H and HCCLM3 cells, whereas its overexpression in Huh-7 cells enhances colony formation, indicating altered proliferative potential. **P < 0.01, ***P < 0.001.

To elucidate SNX16’s function in HCC, stable knockdown models were established in MHCC97H and HCCLM3 cells with high SNX16 expression, and overexpression models in Huh7 cells with low expression (Fig. 2A-D, Supplement Fig. 1C-D). Assessments of SNX16’s impact on HCC proliferation employed CCK-8, EdU incorporation, and clonogenic assays to gauge varied aspects of cell growth dynamics. CCK-8, a prevalent colorimetric viability assay, revealed diminished absorbance with SNX16 knockdown in MHCC97H and HCCLM3 cells, and elevated absorbance upon SNX16 overexpression in Huh-7 cell (Fig. 2E-G), indicative of respective proliferation decreases and increases. EdU labelling, visualized by fluorescence microscopy, confirmed these trends, with reduced EdU-positive cells post-SNX16 silencing and heightened positivity following overexpression (Fig. 2H-L), reflecting DNA replication activity. Clonogenic assays further supported these findings: SNX16 depletion led to fewer, smaller colonies in MHCC97H and HCCLM3 cells, whereas overexpression elicited more extensive colonies in Huh-7 cells (Fig. 2M-O), suggesting perturbed self-renewal and proliferation capabilities. Collectively, these assays robustly demonstrate SNX16’s promotion of HCC proliferation and clonogenic potential, implicating it as a facilitator of tumourigenesis and progression.

### SNX16 supresses HCC-relevant apoptosis

**Figure 3:**
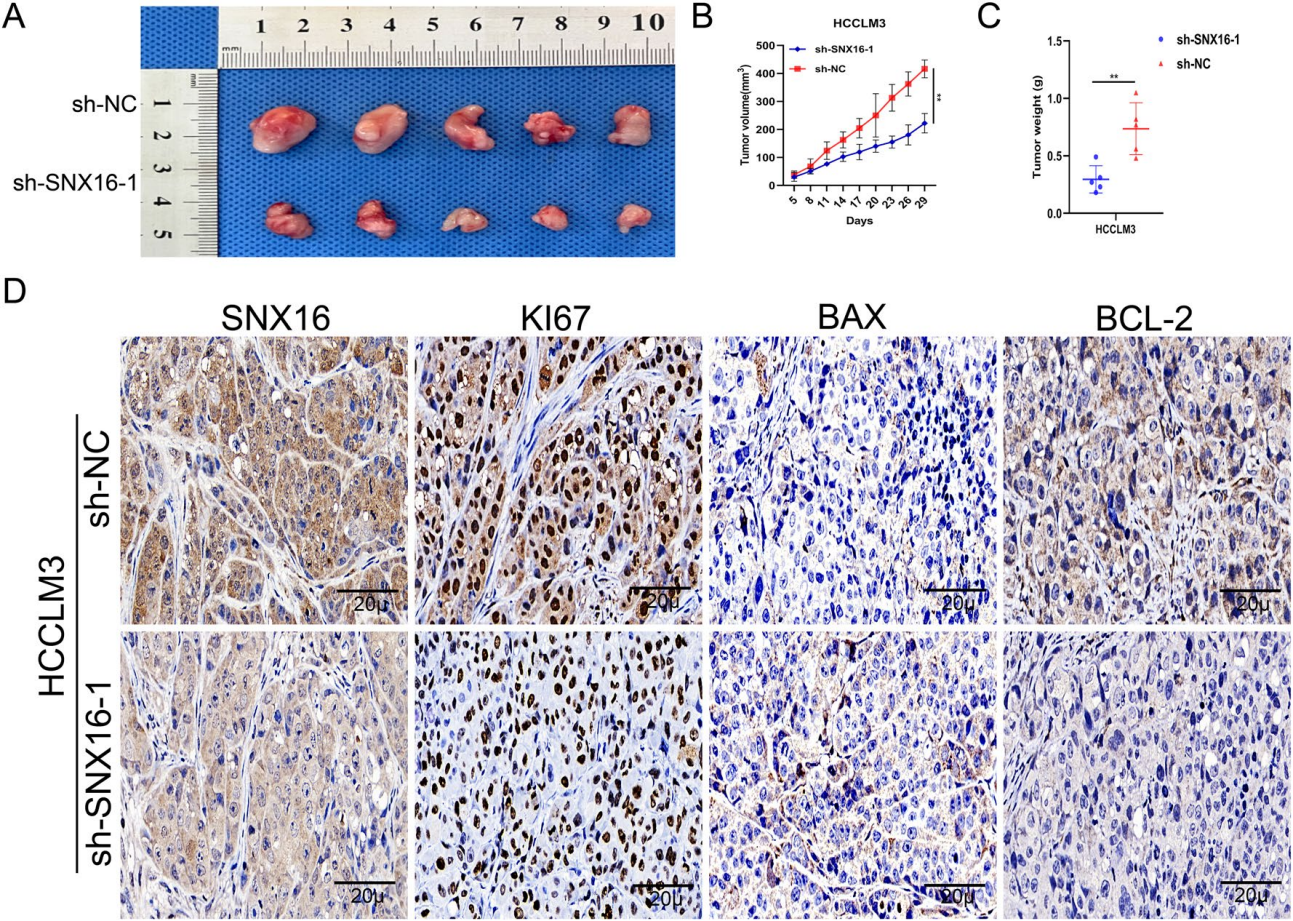
Differential expression of SNX16 modulates apoptosis in HCC cells (**A**-**C**) Flow cytometry was employed to assess apoptosis subsequent to altered SNX16 expression, demonstrating a significant elevation in the combined early and late apoptotic rates in HCCLM3 and MHCC97H cells upon SNX16 knockdown. Conversely, SNX16 overexpression in Huh7 cells resulted in a reduction in the total apoptotic fraction. ns: no sense. *P < 0.05, **P < 0.01. (**D**) Western blot analysis revealed elevated BAX levels but reduced BCL2 and SNX16 expression in HCCLM3 and MHCC97H cells following SNX16 knockdown. Conversely, in Huh7 cells with SNX16 overexpression, a decrease in BAX levels coincided with an upregulation of both BCL2 and SNX16.

Furthermore, Apoptosis analysis by flow cytometry indicates that SNX16 silencing markedly induces overall apoptosis in MHCC97H and HCCLM3 cells. In Huh7 cells, on the other hand, ectopic SNX16 expression significantly reduced the overall apoptosis rate (Fig. 3A-C). The researchers then measured the expression levels of two important regulatory proteins, Bax and BCL2, with strong apoptotic associations. BAX expression was elevated via western blotting among stable SNX16 knockdown MHCC97H and HCCLM3 cells. In contrast, ectopically expressing SNX16 in Huh7 cells resulted in higher expression of BCL-2 (Fig. 3D, Supplement Fig. 1E-F). Collectively, based on the information we have, it seems that SNX16 stimulates cell proliferation and diminishes apoptosis in HCC cells, which is how it causes cancer.

### SNX16 promotes growth of subcutaneous tumours in nude mice

**Figure 4:**
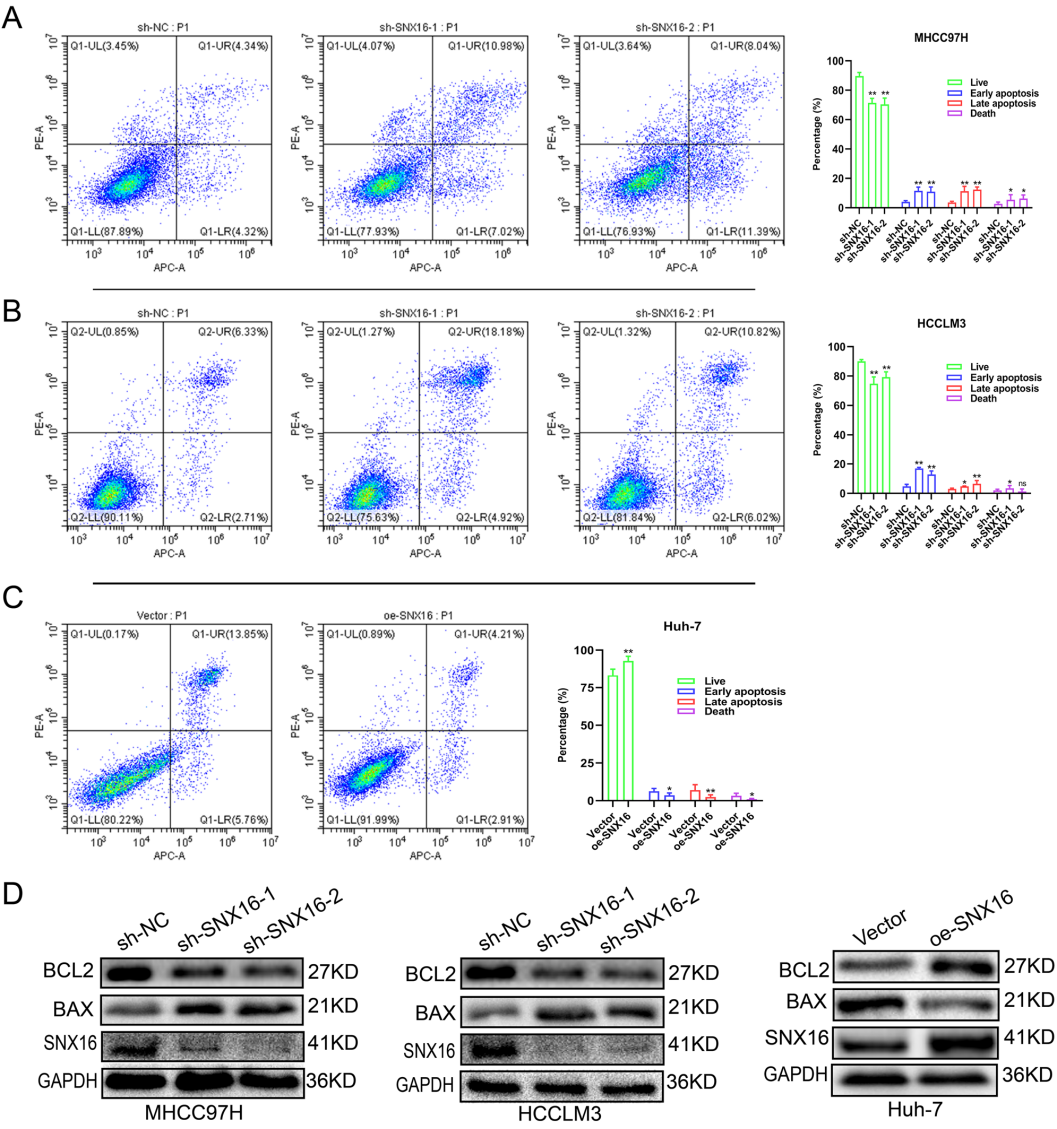
Alterations in SNX16 expression levels influence HCC growth in vitro (**A**-**B**) Representative images of subcutaneous xenograft tumours inoculated with HCCLM3 cells stably silencing SNX16 (N = 5/group). (**C**) Dotplot showing quantitative analysis of tumour weight. (**D**) In HCCLM3 cells, SNX16 expression was suppressed compared to controls. Immunohistochemical analyses of subsequent xenograft tumors revealed altered protein profiles: reduced expression of SNX16, Ki67, and BCL2, coinciding with elevated BAX levels following SNX16 knockdown. Scale bar: 20 μm.

Building upon our in vitro findings, we proceeded to investigate the roles of SNX16 in a live animal setting using subcutaneous xenograft models. We studied the effects of subcutaneous xenografts in naked mice carrying HCCLM3 cells with a stable SNX16 knockdown. The data indicate that SNX16 knockdown in HCCLM3 cells significantly reduced tumour growth and weight compared to negative controls (Fig. 4A-C). Immunohistochemical analyses confirmed the correlations among the expression patterns of Ki67, SNX16, and apoptotic proteins BAX and BCL2, collectively mirroring the status of SNX16 expression. Of particular note, our findings unveiled a compelling scenario whereupon suppression of SNX16 expression led to concurrent reductions in Ki67, BCL2, and SNX16 levels, accompanied by a marked elevation in BAX expression. This shift in protein expression dynamics intimates a potent inhibition of cellular proliferation, thereby implicating a pivotal role for SNX16 modulation in suppressing oncogenic progression in HCC (Fig. 4D).

### SNX16 promotes HCC migration and invasion

**Figure 5:**
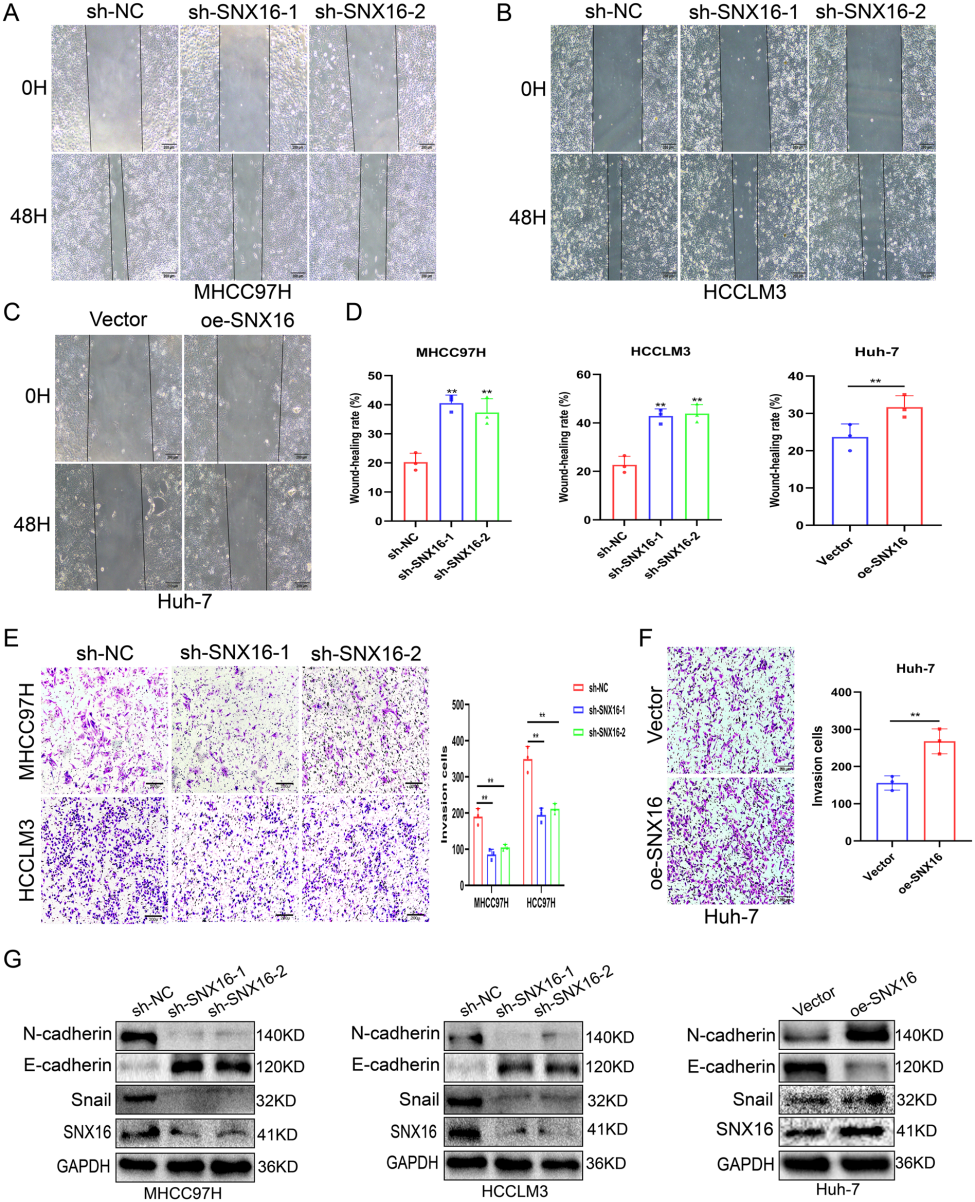
SNX16 promotes HCC migration and invasion (**A**–**D**) The percentage of wound closure, calculated from the change in distance between wound edges, served as a metric for migratory capability. This analysis revealed diminished migration capacity in HCCLM3 and MHCC97H cells following SNX16 knockdown, whereas overexpression of SNX16 in Huh-7 cells resulted in an enhancement in cellular migration. Scale bars: 200 μm. (**E**–**F**) Representative images extracted from invasion assays strikingly depict the modulation of invasive capacities in HCC cells subsequent to alterations in SNX16 expression. Notably, in HCCLM3 and MHCC97H cells with suppressed SNX16 expression, a pronounced reduction in invasion activity was discerned, implying an inhibition of their metastatic potential. In contrast, within Huh-7 cells featuring elevated SNX16 expression, an augmented invasive phenotype was evident, suggesting an increase in cellular aggression concomitant with heightened SNX16 levels. Scale bars: 200 μm. (**G**) Regarding the regulatory effects of SNX16 expression levels on EMT-associated proteins, we found that SNX16 knockdown inhibits the EMT process in HCCLM3 and HCC97H cells, evidenced by reduced expression of Snail and N-cadherin, alongside elevated E-cadherin levels. Conversely, in Huh-7 cells with SNX16 overexpression, EMT is promoted, manifested by decreased E-cadherin and increased levels of Snail and N-cadherin.

Given cancer cells’ migratory and invasive propensities, we examined SNX16’s influence on these traits in HCC cells. Our findings revealed that SNX16 overexpression augmented both invasion and migration capabilities, whereas its knockdown elicited converse effects, suppressing these oncogenic behaviors (Fig. 5A-F). Given the prominent impact of SNX16 on HCC cell proliferation, we further investigated its regulatory effects on the expression of EMT-associated proteins. SNX16 knockdown inhibits EMT in HCCLM3 and HCC97H cells, characterized by lowered Snail and N-cadherin, and E-cadherin elevation, whereas its overexpression in Huh-7 cells fosters EMT, with reduced E-cadherin and heightened Snail/N-cadherin. In summary, these data highlight SNX16 as a pivotal regulator of HCC’s invasive, migratory characteristics, and EMT program, underscoring its central role in disease progression (Fig. 5G, Supplement Fig. 1G-H).

### SNX16 may have potential with AKT signalling pathway

**Figure 6:**
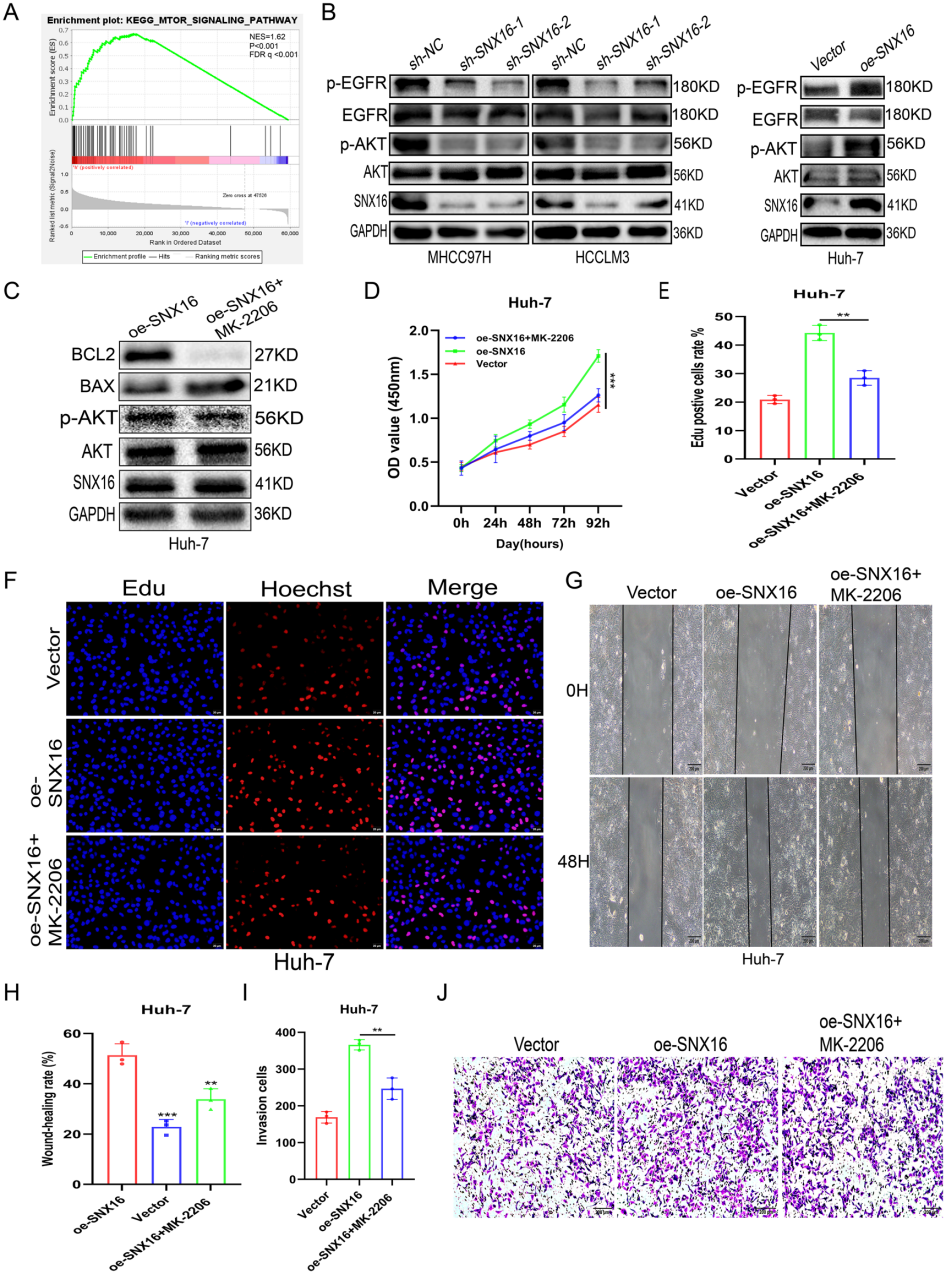
High expression of SNX16 enhances HCC cell invasiveness by activates the AKT signaling pathway (**A**) GSEA has illuminated a positive correlation between SNX16 expression levels, oncogenic processes, and the activation of the AKT/PI3K/mTOR signaling (NES = 1.62, P < 0.001, FDR q < 0.001). (**B**) In HCC cells with upregulated or downregulated SNX16 expression, alterations were observed in the protein expression levels of p-AKT and p-EGFR, whereas the levels of total AKT and EGFR remained unaltered. (**C**) Following treatment with the AKT inhibitor MK2206, a decrease in the protein levels of p-AKT was observed, while the levels of SNX16 and total AKT remained unchanged. (**D**–**F**) Analysis using CCK-8 and Edu assays revealed partial inhibition of HCC proliferation mediated by SNX16 upon treatment with the AKT inhibitor MK-2206, compared to Huh-7 cell overexpressing SNX16. Scale bars: 20 μm. **P < 0.01; ***P < 0.001. (**G**–**J**) Wound healing assays and invasion assays demonstrated a reduction in migration and invasion capabilities of Huh-7 cell, indicative of partial suppression of SNX16-mediated HCC progression under AKT inhibition MK-2206. Scale bars: 200 μm. **P < 0.01; ***P < 0.001.

We evaluated data from the TCGA project to discover how SNX16 modulates tumour growth. Of note, GSEA analysis revealed a compelling association between elevated SNX16 expression and activation of the AKT/PI3K/mTOR signaling cascade in HCC, suggesting a novel regulatory axis (NES = 1.62, p < 0.001, FDR q < 0.001, Fig. 6A). Earlier studies have indicated that increased SNX5 expression in thyroid cells promotes metastatic thyroid tumourigenesis by activating AKT^24^. Reportedly, SNX10 regulates amino acid metabolism through chaperone-mediated autophagy (CMA), influencing mTOR activation, thereby implicating it as a potential therapeutic target and strategy for colorectal cancer intervention^25^. Furthermore, Western blot analysis indicated that SNX16 knockdown in HCCLM3 and MHCC97H cells decreased p-AKT (not AKT) expression. Overexpression of SNX16 in Huh7 cells elevated p-AKT (not AKT) expression (Fig. 6B, supplement Fig. 1I). We examined the impact of the AKT inhibitor MK-2206 (CAS 1032350-13-2) on SNX16-driven migration, invasion, and proliferation in HCC, tracking affiliated protein modifications, revealing unaltered total AKT but markedly reduced p-AKT levels under inhibitor treatment, coincident with BAX elevation and BCL-2 reduction (Fig. 6C). Importantly, SNX16-induced phenotypic shifts, including heightened proliferation and invasive capabilities, are suggested as reversible, given the restoration observed upon AKT inhibition (Fig. 6D-J), thereby implicating AKT pathway mediation in SNX16’s oncogenic effects. Together, the reported findings support that SNX16 drives HCC cell malignancy by triggering the AKT pathway.

### SNX16 promotes HCC progression via EGFR-AKT pathway

**Figure 7:**
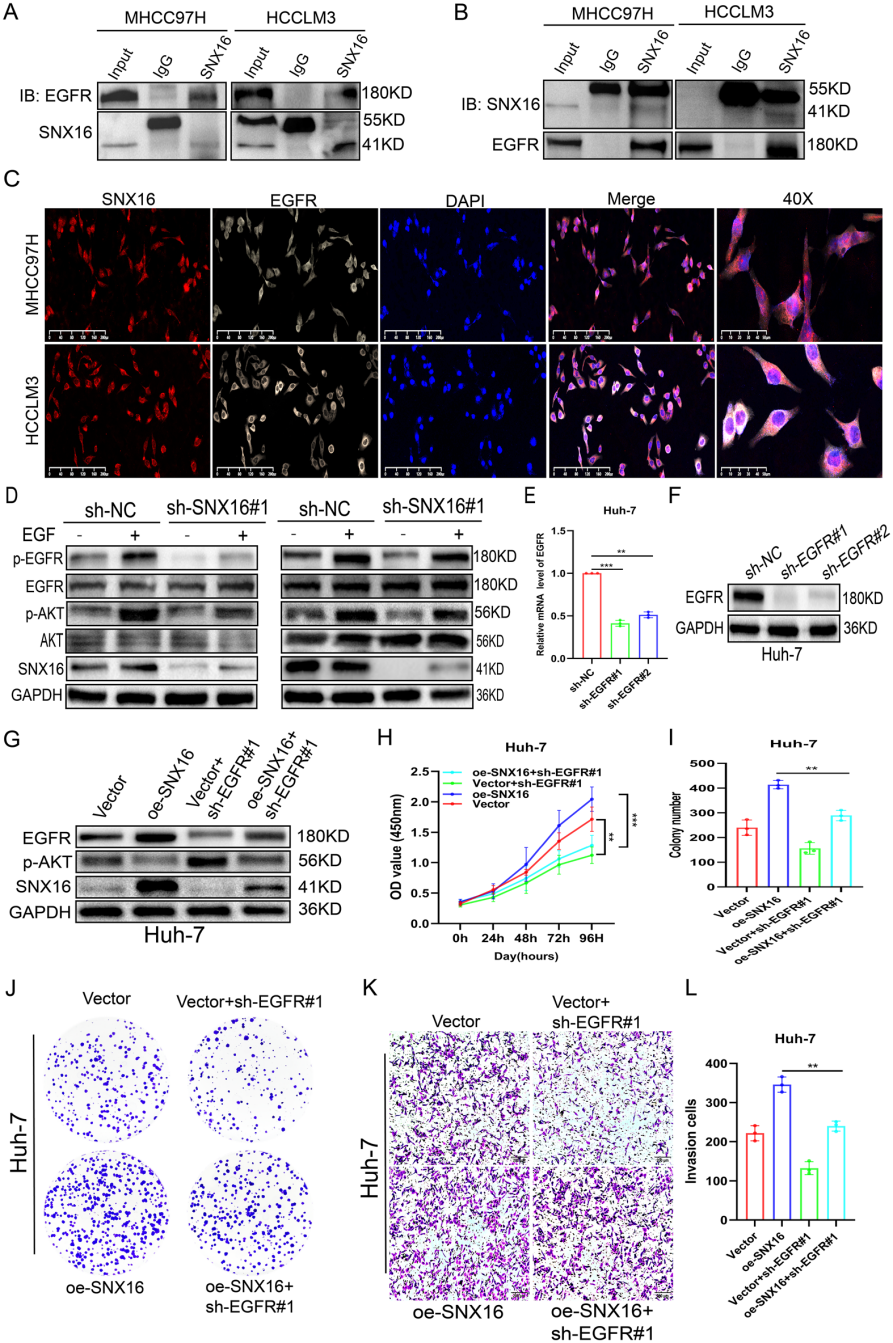
EGFR knockdown attenuates SNX16-overexpressing HCC cell growth and migration (**A**-**B**) Immunoprecipitation assays were conducted using MHCC97H and HCC-LM3 total protein lysates, treated with antibodies against SNX16 and EGFR, or control IgG, revealing a reciprocal interaction between SNX16 and EGFR. (**C**) In MHCC97H and HCC-LM3 cells transfected with SNX16, immunofluorescence staining depicted EGFR (pink) and SNX16 (red), with DAPI (blue) employed for nuclear counterstaining. The experiments uncovered a predominant cytoplasmic co-localization of SNX16 and EGFR, suggesting their interaction primarily occurs within this cellular compartment. Scale bars: 200 μm, 50 μm. (**D**) Following stimulation with 1 µg per milliliter of EGF, p-AKT and p-EGFR levels were found to be elevated in MHCC97H and HCC-LM3 cells where SNX16 had been knocked down, relative to cells with SNX16 knockdown alone, as assessed by protein immunoblotting. Notably, no significant alterations were observed in the expression levels of total AKT and EGFR. (**E**–**F**) The efficacy of EGFR transfection in Huh-7 cells was validated by assessing the expression levels of both mRNA and protein. **P < 0.01; ***P < 0.001. (**G**) In Huh7 cell oe-SNX16 and transfected with sh-EGFR, a restoration in the protein levels of SNX16 and p-AKT was observed. (**H-J**) Following transfection with sh-EGFR, Huh7 cells that subsequently overexpressed SNX16 exhibited a partial recovery in their proliferative capacity, an observation meticulously quantified through CCK-8 assays and colony formation experiments. **P < 0.01; ***P < 0.001. (**K**-**L**) Upon transfection with sh-EGFR in Huh7 cells overexpressing SNX16, a partial restoration of invasive capability was noted, which was meticulously assessed via Transwell assays. Scale bars: 200 μm. **P < 0.01.

High EGFR correlates with elevated HCC risk^26^, many cellular processes are modulated by EGFR, such as cell proliferation, killing and invasion^27,28^. EGFR can activate PI3K/AKT signaling pathway after binding its ligand^29^. We posited that SNX16 modulates the EGFR/AKT axis to advance carcinogenesis and invasion. Experimental evidence confirmed this, showing SNX16 overexpression enhanced EGFR phosphorylation, whereas its depletion attenuated this effect in hepatocellular carcinoma cells (Fig. 6B). We also investigated whether SNX16 had any interaction with HCC-based EGFR. Co-IP assay demonstrated that SNX16 interacts with EGFR (Fig. 7A-B, supplement Fig. 1J). Immunofluorescence (IF) revealed that SNX16 and EGFR colocalised mainly in the cytoplasm of HCCLM3 and MHCC97H cells (Fig. 7C). When treated with epidermal growth factor (EGF), the difference was more pronounced. Notably, control shRNA-treated cells exhibited a significantly stronger response to 1 µg/ml EGF, as evidenced by elevated phosphorylation of EGFR (Tyr1068) and p-AKT, compared to cells depleted of SNX16, highlighting SNX16’s regulatory role in EGFR/AKT signaling (Fig. 7D, Supplement Fig. 1K), but SNX16 didn’t change significantly. These analyses provide evidence that SNX16 enhances EGFR signalling by binding to EGFR.

To clarify the role of EGFR in HCC, we assessed sh-EGFR knockdown efficiency in Huh-7 cells (Fig. 7E-F). Our results demonstrate that EGFR silencing abrogates SNX16-induced p-AKT upregulation. Moreover, it partially reversed the heightened invasion, migration, and proliferation stimulated by SNX16 overexpression, implicating EGFR as a crucial mediator of SNX16’s oncogenic actions (Fig. 7G-L, Supplement Fig. 1L-M). Collectively, our results imply that SNX16 fosters HCC invasiveness, migratory capacity, and proliferative potential through the activation of the EGFR-AKT signaling cascade.

## Discussion

**Figure 8:**
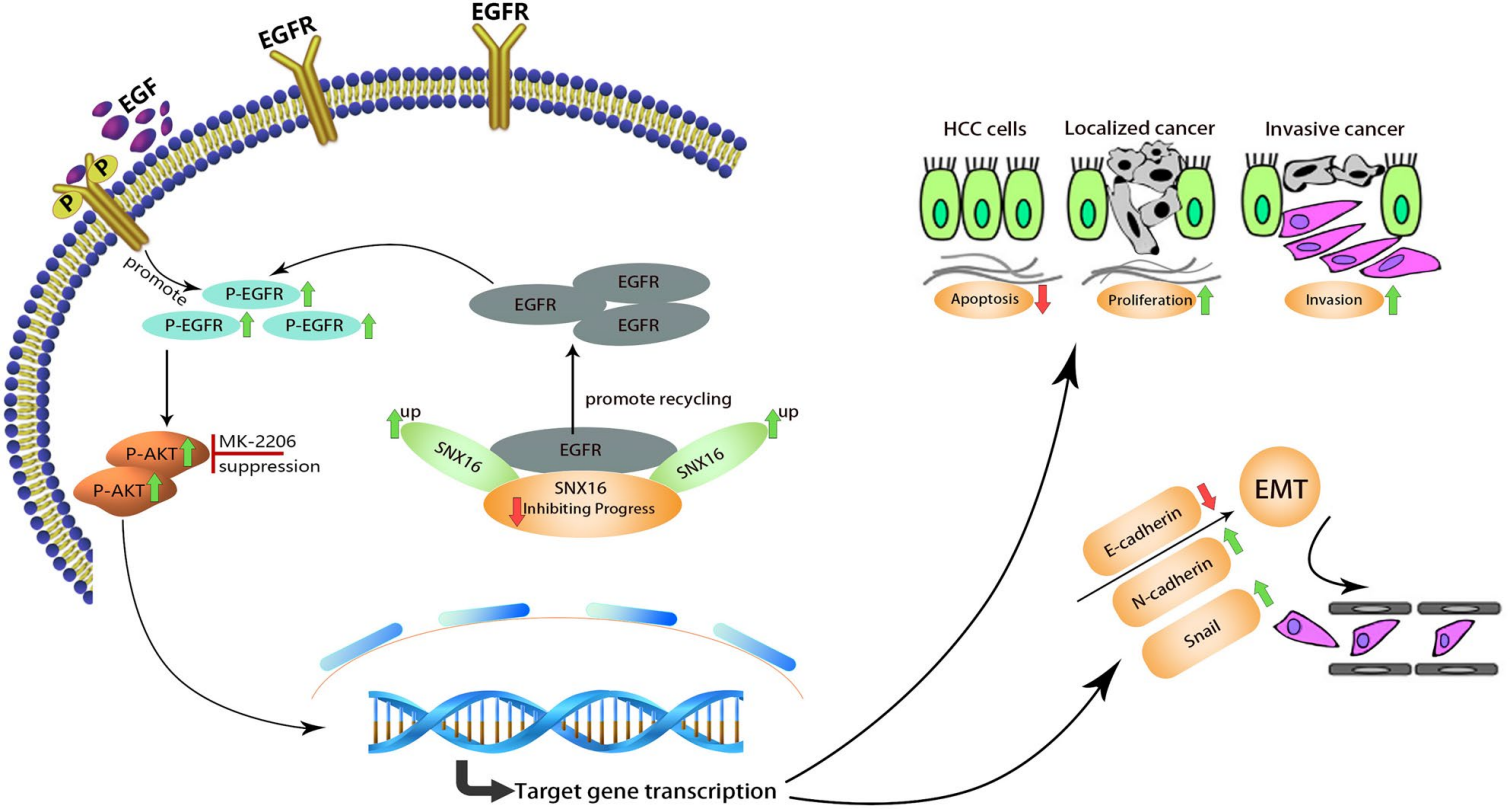
(**A**) A model elucidating the mechanism by which SNX16 fosters HCC progression through the activation of the EGFR/AKT signaling axis.

Our study reveals elevated SNX16 expression as a hallmark of disease progression, fueling HCC proliferation and invasion. SNX16 levels also correlate with patient prognosis. Mechanistically, SNX16 stimulates the EGFR-AKT axis to drive tumorigenicity. Notably, silencing EGFR mitigates the pro-oncogenic effects imparted by SNX16 overexpression in HCC cell, underscoring a critical interplay in disease progression. This investigation emphasizes the pivotal role of SNX16 in the pathogenesis of HCC, positioning it as a crucial regulatory node and a promising therapeutic target for impeding disease progression (Fig. 8A).

Endocytic processes, known to influence transformed cell behavior such as growth and dissemination^30^, have garnered attention for the SNX family, particularly regarding their role in endocytosis^11,31^. Among these, SNX16 stands out, with accumulating evidence linking it to endocytic pathways and the internalization of membrane-associated lipids and proteins^11^. In this investigation, we discovered that SNX16 increased HCC cell proliferation but inhibited apoptosis and promoted subcutaneous tumour development within nude mice HCC cells. In addition, recent findings have revealed that endocytosis is an indispensable element of cell mobility^32^. Our study showed that overexpressing SNX16 also increased migration, invasion and EMT expression. Conversely, SNX16 knockdown had an inhibitory effect on malignancy. Notably, these factors typically facilitate cellular dedifferentiation and enhance migratory and invasive capabilities during EMT. Under normal conditions, E-cadherin aids in preserving tight junctions between cells, and its downregulation is often regarded as a hallmark of EMT initiation. Multiple malignancies have been linked to SNX16, including colorectal cancer^13^ and renal cell carcinoma^9^. Overexpression of SNX16 in bladder cancer patients’ blood cells^12^. Alternative Splicing Sequence Enriched Tag Sequencing to identify the important role of SNX16 in melanoma development^33^. Contrarily, earlier work employing a mouse xenograft model demonstrated that elevated SNX16 expression in MCF-7 breast cancer cells inversely suppressed tumor expansion and migratory activity^34^. Tian et al. show KLF9-SNX5 promoter binding accelerates ccRCC progression and poor outcomes^35^. Our prior work implicates HOXB9 in colorectal oncogenesis^36,37^; investigating HOXB9-SNX16 interactions is warranted. Our study, through in vitro and in vivo gain- and loss-of-function assays, has implicated SNX16 as an oncogenic driver in HCC. To fully decipher the molecular pathways through which SNX16 governs HCC cell proliferation and to assess its viability as a therapeutic intervention point, further rigorous inquiries are imperative.

A review of the literature indicates that SNXs play a pivotal role in regulating key signaling pathways implicated in oncogenesis, notably including the EGFR signaling^38^. SNXs, including SNX5 in HCC via ERK1/2^22^. Teasdale et al. demonstrated that depleting SNX3 blocks EGFR degradation and activates ERK1/2 and Akt^39^. AKT central to EGFR-mediated cell migration^40^. SNX16 is known to help regulate Epidermal Growth Factor-induced signalling^41^. This led us to further speculate whether the EGFR/AKT signaling cascade represents a pivotal pathway governed by SNX16 in HCC cells. Furthermore, the results by confocal and immunoprecipitation assays showed that EGFR and SNX16 mostly bind to each other in the cytoplasm. The EGFR, a member of the ErbB oncogene family, plays a crucial role in tumorigenesis and the advancement of malignant tumors^42^. EGF is a high affinity ligand of EGFR^43^. The stimulation of the EGF/EGFR signaling cascade accelerates cancer progression by fostering metastases, triggering angiogenesis, facilitating cell invasion and multiplication, and concurrently hindering apoptotic cell death^44^. It was revealed that p-EGFR (Tyr1068) and p-AKT were partially restored when EGF was added to control shRNA cells in comparison to SNX16 knockdown HCC cells. Our investigation reveals that SNX16 regulates disease malignancy by selectively tuning EGFR and AKT phosphorylation, without altering total protein levels. Through SNX16 manipulation, the balance of EGFR trafficking—endocytosis, dephosphorylation, recycling, or degradation—is disrupted, impacting EGFR activation via phosphorylation shifts. Downstream, AKT activity is differentially modulated, potentially implicating specific kinase/phosphatase regulation. This demonstrates precise control over phosphorylation dynamics within oncogenic pathways, preserving protein homeostasis, and highlighting the complexity of intracellular signal regulation.

In terms of functional rescue, knockdown of EGFR or AKT inhibitors (MK-2206(CAS1032350-13-2)) partially abolished the cell proliferation, migration and invasiveness triggered by SNX16 overexpression. Studies have revealed that an elevated level of SNX5 expression in NR6/EGFR cells is linked with a diminished rate of EGFR protein breakdown^45^. Losing SNX5 causes internalised EGFR to stabilise and drive thyroid carcinogenesis^24^. Evidence suggests a positive feedback mechanism exists between SNX5 and EGFR in HCC cells, wherein SNX5 suppression reduces EGFR surface expression and enhances its cytoplasmic internalization upon EGF stimulation^22^. Similarly, SNX16 steers EGFR towards the endosomal compartment, thereby finetuning EGF-induced cellular signaling^41^. SNXs regulate EGFR transport and membrane localization, impacting receptor activation and disease. Our work identifies a knowledge gap in SNX16-EGFR interaction mechanisms, an underexplored area crucial for future studies to elucidate this complex regulatory system.

## Conclusion

Elevated SNX16 expression in HCC and a robust correlation with unfavorable patient survival outcomes. Targeting the hyperproliferative phenotype of HCC cells through inhibition of the EGFR/AKT signaling axis emerges as a novel therapeutic strategy, implicating SNX16 as a pivotal regulator.

## Materials and methods

### Data collection and organization

The first step was to quantify the SNX16 mRNA expression in HCC using the online databases TCGA (www.tcga.org/). Inclusion criteria comprised patients with a confirmed diagnosis of HCC. Exclusions were: (1) cases lacking clinical follow-up data, (2) those with unknown survival times or status, and (3) duplicate patient records. Ultimately, 371 HCC patients and 50 normal samples, accompanied by comprehensive clinical data including age, gender, tumor grade, stage, and TNM classification, were selected for further analyses (Table 1). Expression alterations between tumor and matched normal tissues were analyzed using the R package “limma”. Patients were stratified into high and low SNX16 expression cohorts based on an optimized cutoff derived from clinical data, including survival time and status. Thereafter, the associations between SNX16 expression and disease-free survival (DFS) and overall survival (OS) in HCC were assessed utilizing the R packages “survminer” and “survival”, providing insights into SNX16’s prognostic relevance.

### Cell culture and lines

Four HCC cell lines (MHCC97H, HCCLM3, HepG2, Huh-7) and the normal cell line THLE-2 were obtained from the Cell Bank of the Chinese Academy of Sciences (Shanghai, China) and Shanghai Hongshun Biotechnology Co., respectively. Cultivation was conducted in DMEM enriched with 1% penicillin–streptomycin (Beyotime, Shanghai, China) and 10% fetal bovine serum (FBS, Gibco, Thermo Fisher Scientific, USA) under a humidified atmosphere containing 5% CO_2_ at 37 °C.

### Procurement of tissue specimens and compilation of clinical records

Human HCC and adjacent non-tumor tissues (n = 8) were surgically procured from the Second Affiliated Hospital of Nanchang University, promptly snap-frozen in liquid nitrogen, and preserved at− 80 °C for subsequent analysis. Ethical approval was obtained from the Institutional Review Board of Nanchang University, and all participants provided written, informed consent in accordance with the Declaration of Helsinki^46^. Ensuing procedures adhered strictly to approved ethical guidelines.

### Plasmids and lentivirus production

To induce SNX16 knockdown, HCCLM3 and MHCC97H cells were transduced with a lentiviral vector encoding an optimized SNX16-targeting shRNA (sh-SNX16; LV-SNX16-RNAi) procured from GeneChem (Shanghai, China), while a control vector (sh-NC) served as a negative control. For SNX16 overexpression, LV-SNX16-Huh-7 vectors were utilized, alongside LV-NC-Huh-7 as additional controls. An EGFR-targeting lentiviral shRNA plasmid was also sourced from GeneChem. Target sequences are detailed in Supplementary Table S1. Transfections adhered to supplier protocols, briefly involving incubation of HCC cells with 1 × 10^6 transducing units of recombinant lentivirus in the presence of 6 μg/ml Polybrene (Sigma).

### Quantitative real-time PCR (qRT-PCR)

Total RNA extraction from HCC tissues, adjacent normal tissues, and cell lines was performed using TRIzol reagent (Invitrogen, USA). cDNA synthesis was procured with the RevertAid First Strand cDNA Synthesis Kit (Thermo Fisher, USA) following RNA denaturation. QRT-PCR was subsequently conducted using a SYBR Green PCR Master Mix Kit (Takara, China). Expression levels of target genes, normalized against GAPDH, were quantified relative to control samples. Primer sequences employed are listed in Supplementary Table S1.

### Western blotting

Cells were resolved on 10% SDS–polyacrylamide gels and transferred onto nitrocellulose membranes. Primary antibodies were incubated overnight at 4 °C, followed by secondary horseradish peroxidase-conjugated antibodies the next day. Protein bands were visualized using Pierce’s enhanced chemiluminescence reagent (Rockford, IL, USA). Antibody details are listed in Supplementary Table S2.

### Cellular proliferation capacity

Cell proliferation was assessed using a Cell Counting Kit-8 (CCK8) from Bimake (USA), following the manufacturer’s protocol. For the Edu assay, cells were harvested post-transfection and plated at a density of 2 × 10^4 cells/ml onto 96-well plates according to the BeyoClick^™^ EDU-488 Kit instructions (Beyotime, Shanghai). Colony formation assays entailed plating cells in six-well plates for two weeks. Colonies were subsequently fixed in 4% paraformaldehyde (pH 7.4) and stained with Giemsa for 30 min.

### Wound healing assay

Post-lentiviral transfection, cells were deposited into 6-well plates. Upon reaching confluence, a linear scratch was made using a 200 µl pipette tip to create a wound. Following two washes with PBS to remove debris, cells were held at 37 °C under 5% CO_2_. Wound closure was monitored periodically using an Olympus IX73 microscope, and images were processed with Adobe Illustrator CC 2017 (NIH) to analyze the wounded area.

### Transwell assay

Cells (3 × 10^4 cells/mL in 200 μL serum-free media) were seeded onto Matrigel-coated (BD Biosciences, NJ) transwells with 8 μm pores. Lower chambers contained 800 μL of 20% FBS-supplemented DMEM. Post 24–48 h incubation, non-invading cells were gently removed. Invasion was quantified by staining and counting cells in five random fields of view.

### Apoptosis analysis

Cells were washed twice with phosphate-buffered saline (PBS) and lysed with EDTA-free trypsin. Apoptotic cells were discerned using flow cytometry employing the Annexin V-APC/PI Apoptosis Detection Kit (KeyGen, Nanjing, China; #KGA1030-50), following the manufacturer’s protocol. Each experiment was triplicated to ensure reproducibility.

### Co-immunoprecipitation (Co-IP) assay

For immunoprecipitation, total protein was retrieved from HCCLM3 and MHCC97H cells using lysis buffer fortified with protease and phosphatase inhibitors. Cell lysates were nurtured overnight at 4 °C with anti-SNX16 antibody (Santa Cruz, MA, USA) or IgG (negative control), followed by 4 h incubation with Protein A/G-agarose beads (Thermo Scientific, Waltham, MA, USA) under gentle agitation. Beads were then boiled, washed, and resuspended in PBS and 5 × loading buffer. SDS/PAGE and electroblotting were employed for protein detection.

### Subcutaneous xenograft model

All animal experiments were authorized by the Animal Care and Use Committee of Nanchang University, adhering strictly to the Guide for the Care and Use of Laboratory Animals and the legislation of the People’s Republic of China governing laboratory animal usage and welfare (Approval No. NCU-2023-0826). Compliance with the ARRIVE guidelines for mouse experiments is also affirmed, ensuring ethical and rigorous scientific practice^47^. Female BALB/c nude mice, aged 4 weeks, were procured from the Laboratory Animal Center of Nanchang University. Random allocation into sh-NC and sh-SNX16#1 groups ensued, with subcutaneous injection of 100 μL stable HCCLM3 cells (2 × 10^7 cells/mL) transfected with respective constructs. Tumor growth was monitored by measuring volume (volume = length × width^2 × 0.5) every 5 days. On day 26 post-inoculation, mice were euthanized, tumors were excised, and their weights recorded.

### Immunohistochemistry (IHC)

Eight paired HCC tumor and adjacent non-tumor tissue samples underwent qRT-PCR, IHC, and Western blot assessments. IHC protocol adhered to standard steps: deparaffinization, xylene treatment, Tris/EDTA (pH 9.5) antigen retrieval, and microwave heating for 20 min.

### Immunofluorescence (IF) staining

Cells were fixed with 4% paraformaldehyde, permeabilized with 0.5% Triton X-100, and blocked in 1% BSA for 20 min at room temperature. Primary antibodies were administered overnight at 4 °C, followed by three PBS rinses. Cellular nuclei were stained with a DAPI kit, and secondary antibodies were incubated for fluorescence visualization of primary antibody binding. Imaging was conducted using inverted microscopy.

### Gene set enrichment analysis (GSEA)

Differential analysis between SNX16high (n = 185) and SNX16low (n = 185) groups employed GSEA v4.0 (www.gsea-msigdb.org/gsea/index.jsp). Using SNX16 expression for 1000 permutations, pathway regulation was elucidated with criteria: NES > 1, P < 0.05, FDR q < 0.25.

### Statistical analysis

Data analyses were conducted using GraphPad Prism 8.0 (GraphPad, San Diego, USA). Unpaired Student’s t-tests assessed differences between two groups, whereas paired t-tests compared SNX16 expression in tumor and adjacent normal tissues. Analysis of Variance (ANOVA) was employed for multiple group comparisons. The Spearman correlation assessed variable relationships. Survival analyses were undertaken using the log-rank test, with statistical significance set at p < 0.05 throughout.

### Supplementary Information

Supplementary Figure 1.

Supplementary Table S1.

Supplementary Table S2.

## Data Availability

The datasets generated and analyzed within this study are accessible in TCGA repository (https://portal.gdc.cancer.gov/), GSEA (https://www.gsea-msigdb.org/gsea/index.jsp) and TIMER2.0 (https://cistrome.shinyapps.io/timer/) databases. Protein sequences, DNA and RNA sequences, etc. was not included in this study. Data in all databases are open and freely available.
