# Supplementary material for: SNX16 is required for hepatocellular carcinoma survival via modulating the EGFR-AKT signaling pathway
Source: Sci Rep. 2024 Jun 7;14:13093. doi: 10.1038/s41598-024-64015-6 (PMC11161632; doi:10.1038/s41598-024-64015-6)
Supplement: Supplementary file 2 — Supplementary Table S1. [file 41598_2024_64015_MOESM2_ESM.docx]

Supplementary Table 1. Primers, shRNA target sequences

| Name | Sequences |
| --- | --- |
| Primers for real-time PCR: |  |
| SNX16 sense | 5'-TGCACTTGAGGTTGATCAAGATGTC-3' |
| SNX16 antisense | 5'-CAGCATCATATGCCACTTCTGCTAC-3' |
| EGFR sense | 5ʹ-AGGCACGAGTAACAAGCTCAC-3ʹ |
| EGFR antisense | 5ʹ- ATGAGGACATAACCAGCCACC-3ʹ |
| AKT sense | 5’-ATGACTTCGGAGATGAACAA-3’ |
| AKT antisense | 5’-CTACTTGAGGTTCCAGTAGC-3’ |
| GAPDH sense: | 5'-AGAAGGCTGGGGCTCATTTG-3′ |
| GAPDH antisense: | 5'-AGGGGCCATCCACAGTCTTC-3′ |
| The target sites of shRNA: |  |
| sh-SNX16#1 sense | 5’-GATCCCCGAAAGCTCTGTTCGATTCAAGAGATCGAACAGAGCTTTCGTTTTTGGAAA-3’ |
| sh-SNX16#1 antisense | 3’-CTAGGGCTTTTCGAGACAAAGCTAAGTTCTCTAGCTTGTCTCGAAAACAAAAACCTTTT-5’ |
| sh-SNX16#2 sense | 5'-GAAAGCUCUGUUGACAUUAAAATCATCAAGAGAATCACATCTGTGTATCCTCTTTTTTG-3′ |
| sh-SNX16#2 antisense | 5'-CTTTCGTGTCTTCTGTTTTTAGTAGTTCTCTTAGTGTAGACACATAGGAGAAAAAAC-3′ |
| sh-NC sense | 5'-GATCCGTTCTCCGAACGTGTCACGTAATTCAAGAGATTACGTGACACGTTCGGAGAATTTTTTC-3′ |
| sh-NC antisense | 5'-AATTGAAAAAATTCTCCGAACGTGTCACGTAATCTCTTGAATTACGTGACACGTTCGGAGAACG-3′ |
| sh-EGFR sense | 5'-CGCAAAGTGTGTAACGGAATA-3′ |
| sh-EGFR antisense | 5'-CATCAGTGGCGATCTCCACAT-3′ |
