# Supplementary material for: SNX16 is required for hepatocellular carcinoma survival via modulating the EGFR-AKT signaling pathway
Source: Sci Rep. 2024 Jun 7;14:13093. doi: 10.1038/s41598-024-64015-6 (PMC11161632; doi:10.1038/s41598-024-64015-6)
Supplement: Supplementary file 3 — Supplementary Table S2. [file 41598_2024_64015_MOESM3_ESM.docx]

Supplement Table S2. Antibodies used in this study

| Antibody | catalog | Dilution | Company |
| --- | --- | --- | --- |
| For Western blotting | | | |
| SNX16 | TA890009 | 1:800 | Origene |
| EGFR | ab52894 | 1:500 | Abcam |
| p-EGFR | ab40815 | 1:1000 | Abcam |
| p-AKT | ab38449 | 1:500 | Abcam |
| AKT | ab8805 | 1:1000 | Abcam |
| BAX | 50599-2-Ig | 1:800 | Proteintech |
| BCL2 | 68103-1-Ig | 1:800 | Proteintech |
| Snail | ab216347 | 1:1000 | Abcam |
| E-cadherin | ab40772 | 1:800 | Abcam |
| N-cadherin | ab76011 | 1:800 | Abcam |
| IgG | sc-2003 | 1:1000 | Santa Cruz |
| Secondary antibody | HRP conjugated goat anti-rabbit IgG | 1:8000 | Proteintech |
| Secondary antibody | HRP conjugated goat anti-mouse IgG | 1:8000 | Proteintech |
| For Immunohistochemistry |  |  |  |
| SNX16 | TA890009 | 1:50 | Origene |
| Ki67 | 27309-1-AP | 1:50 | Proteintech |
| Secondary antibody | Envision kit (HRP, rabbit/mouse, DAB+) | Ready-to-use | DAKO |
| For Immunofluorescence staining |  |  |  |
| SNX16 | sc-271260 | 1:40 | Santa Cruz |
| EGFR | ab52894 | 1:50 | Abcam |
| Secondary antibody | Alexa Fluor 555 anti-mouse IgG | 1:50 | Invitrogen |
| Secondary antibody | Alexa Fluor 594 anti-rabbit IgG | 1:50 | Invitrogen |
